# Building resiliency: a cross-sectional study examining relationships among health-related quality of life, well-being, and disaster preparedness

**DOI:** 10.1186/1477-7525-12-85

**Published:** 2014-06-09

**Authors:** Monica E Gowan, Ray C Kirk, Jeff A Sloan

**Affiliations:** 1 Department of Health Sciences Research, Mayo Clinic, 200 First St SW, Rochester, MN, USA; 2 School of Health Sciences, College of Education, University of Canterbury, Christchurch, New Zealand

**Keywords:** Earthquake, Evacuation, Hazards, Health promotion, Health outcomes, Prevention, Integrative disaster resilience, Risk perception, Self-management, Tsunami

## Abstract

**Background:**

Worldwide, disaster exposure and consequences are rising. Disaster risk in New Zealand is amplified by island geography, isolation, and ubiquitous natural hazards. Wellington, the capital city, has vital needs for evacuation preparedness and resilience to the devastating impacts and increasing uncertainties of earthquake and tsunami disasters. While poor quality of life (QoL) is widely-associated with low levels of engagement in many health-protective behaviors, the relationships among health-related quality of life (HrQoL), well-being, and preparedness are virtually unknown.

**Methods:**

We hypothesized that QoL and well-being affect household evacuation preparedness. We performed a quantitative epidemiologic survey (cross-sectional design) of Wellington adults. Our investigation assessed health-promoting attributes that build resiliency, conceptualized as health-protective attitudes and behaviors. Multidimensional QoL variables were measured using validated psychometric scales and analyzed for associations with evacuation preparedness, and we determined whether age and gender affected these relationships.

**Results:**

We received 695 survey responses (28.5% response rate; margin of error ±3.8%; 80% statistical power to detect true correlations of 0.11 or greater). Correlational analyses showed statistically significant positive associations with evacuation preparedness for spiritual well-being, emotional well-being, and life satisfaction. No associations were found for mental health, social well-being, or gender; physical health was weakly negatively associated. Evacuation preparedness increased with age. Regression analyses showed that overall health and well-being explained 4.6-6.8% of the variance in evacuation preparedness. Spiritual well-being was the only QoL variable that significantly and uniquely explained variance in preparedness.

**Conclusions:**

How well-being influences preparedness is complex and deeply personal. The data indicate that multidimensional readiness is essential, and meaningfulness is an important factor. Inadequate levels of tangible preparedness actions are accompanied by gaps in intangible readiness aspects, such as: 1) errors in perceived exposure to and salience of natural hazards, yielding circumscribed risk assessments; 2) unfamiliarity with the scope and span of preparedness; 3) underestimating disaster consequences; and 4) misinterpreting the personal resources required for self-managing disaster and uncertainty. Our results highlight that conceptualizing preparedness to include attitudes and behaviors of readiness, integrating well-being and meaningfulness into preparedness strategies, and prioritizing evacuation planning are critical for resiliency as a dynamic process and outcome.

## Introduction

Life-threatening and life-changing natural disasters are prevalent throughout the Pacific Rim and Indian Ocean area. Asia-Pacific leads the world with the greatest number of disasters, highest associated mortality, and greatest economic losses this millennium [1]. New Zealand is a heightened disaster “riskscape” [2] because of its isolated location (roughly 1,500 km east of Australia), over 15,000 km of coastline, small landmass (<270,000 km^2^), and omnipresent vulnerability to natural hazards [3-5], including seismic hazards arising from local, regional, and trans-Pacific sources [6-8].

In fact, a complex, long-lasting series of damaging earthquakes (the “Canterbury earthquake sequence”) with extremely high ground motions [9-11] struck Christchurch and the Canterbury Region of New Zealand’s South Island over a 2-year period during the tenure of this study, claiming scores of lives [12]. An estimated 900 commercial buildings in the central business district and more than 30,000 residential properties were declared total losses [13,14]. Tens of thousands were forced from their homes and workplaces [15]. These events strained utility infrastructure [16] and the health sector response capacity [17,18] and dramatically affected the national economy [19,20]. Seismicity is ongoing in Canterbury [9] and elsewhere in New Zealand; strong earthquakes struck the Wellington and Marlborough Regions (the “Cook Strait Earthquakes”) in mid-2013 and the Wellington and Manawatu-Wanganui Regions (the “Eketahuna Earthquake”) in early 2014 [21].

The coastal, capital city of Wellington, the geographic focus of this investigation, has a long history of earthquakes and tsunamis [22,23]. Most of the regional population and the nation’s governance are vulnerable to these seismic threats [24]. In addition to mass casualties [25], damage to homes, utilities, services, and the economy could result in mass evacuations and extreme hardship [26-28] from the national to household level.

Devastating earthquakes and tsunamis also profoundly and persistently affect human health [29] and quality of life (QoL) [30,31]. Poor psychological and economic well-being can induce cognitive disruption [32,33] and prolong recovery from deep hardship by creating comorbid and chronic health effects [33-37]. Dislocation and internal displacement can induce disorientation and disconnection [38], and evacuees may have up to twice the rate of illness than nonevacuees affected by disaster [39].

Disasters are thus ultimately health outcomes, a function of how people are affected by or respond to the ambient riskscape. As in preventive medicine, adverse disaster outcomes therefore are somewhat preventable. Although avoiding natural hazards may be impossible, preparation can reduce vulnerabilities and minimize exposures to calamitous outcomes [40,41].

### Evolving paradigms in disaster management and research

Disaster risk reduction (DRR) [42] focuses on building an engineering and systems-wide capacity for dynamic adaptation [43] through risk management. DRR is often equated with *minding the risk* and, often by extension, *resilience*. The meaning of resilience, however, is evolving with time and application [44-46]. We argue that minding the risk and resilience go beyond managing hazards, vulnerability, and exposure; a framework for self-managing consequences is essential [46,47]. Extreme events require robust decision-making capacities and deep personal resources, especially when displacement unexpectedly occurs [48,49]. Moreover, people differ in how they perceive and process their risks affectively and cognitively [50,51].

We conceptualize disaster resilience as a process and outcome that embodies disaster prevention. We consider personal resiliency to be a dynamic and multidimensional state of well-being and readiness that manifests as a fundamental awareness (“the threat is present”), acceptance of potential loss (“it can happen to me”), empowering, protective attitudes (“I have resources and choices”), and engagement in preventive actions (“I am actively building personal resources and adaptive capacities”). By cultivating well-being and intentionally engaging in preparedness, readiness becomes more than risk management; it is an integrative, fluid, and health-promoting state (“I am as well as I can be”) that facilitates adaptive postdisaster trajectories [52,53]. This state of positive health can be available and accessible, regardless of external circumstances [54].

Preventing disaster by reducing risks *and* enhancing protective processes is now a global priority [55-57]; the United Nations currently proposes to name the post-2015 framework for disaster risk reduction “*Managing Risk to Achieve Resilience*.” These needs are recognized by New Zealand civil defense and emergency management [58], and Wellington recently was named 1 of 10 partner cities worldwide for the City Resilience Profiling Programme under the United Nations Human Settlements Programme [59].

Historically, disaster research in the biomedical and social sciences has largely targeted risk reduction via the traditional focus on disease and diminished capacities; for example, the role of specific vulnerabilities or risk factors and their pathogenic relationships with morbidity and mortality (risk/deficit approach) have been examined [37,60,61]. Research has also addressed the amplification of vulnerability among those with chronic disease and disability [62-64]. Appropriately, previous work in promoting disaster preparedness has focused strongly on overcoming medical, social, and demographic barriers to preparedness actions [40,65-67].

However, resiliency research increasingly uses a biopsychosocial model (strength/asset approach) [68-70] to evaluate the influence of health-enhancing or protective factors (health management resources) on recovery trajectories [71-73]. Indeed, multidimensional, salutogenic (wellness) paradigms [74,75] have long been harmonious with definitions from the World Health Organization (WHO) for health and QoL [76,77]. They are also consistent with research findings showing that altruistic and existential attitudes, intrinsically motivated goals, and salutary behaviors support growth and build resiliency after traumatic events [78-80]. Further, they are complementary with research into the cognitive and social processing of preparedness messages [48-51], the effect of experience on hazard adjustments [81], current models of protective action decision-making [48], and an emerging shift toward a health-promoting, community-level emphasis on “what to do about risk” [82].

### Need to strengthen the evidence base

A compelling need remains for evidence-based research [82-87]. In New Zealand, national baseline data on health, QoL, well-being, and emergency preparedness can be used to determine the status of health management resources and capacity for resiliency-building at the population level [88-92]. Nevertheless, associations among QoL and well-being (health-protective attitudes) and disaster preparedness (health-protective behavior) are unknown, and only one study has examined the role of quality of life in disaster preparedness [93]. To our knowledge, relationships between these affective and behavioral constructs are unexplored elsewhere, nor have they been evaluated in the context of earthquake or tsunami evacuation.

We assessed the prevalence of health-related QoL (HrQoL) and global life satisfaction (subjective well-being) by using validated psychometric instruments and measured the level of engagement in disaster preparedness, with a specific focus on evacuation preparedness. We determined associations among domains of well-being and preparedness behaviors and also collected data on cognitive and affective risk perceptions for earthquake and tsunami disaster. Our results expand the evidence base on QoL and well-being to support efforts to 1) meet emergency management needs in Wellington and 2) promote continued and effective resiliency-building in all communities at risk.

## Methods

We hypothesized that adults with higher quality of life will exhibit higher levels of household preparedness for earthquake and tsunami. We developed measureable attributes of resilience by examining QoL constructs and operationalizing selected indicators of multidimensional well-being and disaster preparedness. The framework [94-99] used to assess health management resources is presented in Table 1. We selected a cross-sectional design, with a random sample drawn from the general adult population. We used a quantitative epidemiologic survey instrument to measure the prevalence of and relationships among key variables. The survey contained 56 questions obtained or derived from validated psychometric scales and QoL instrument databases [100,101], social science surveys on disaster in New Zealand, Australia, and the United States [102-105], and questions developed specifically for this study; demographic questions from the New Zealand census also were included [106]. Ethics notifications and approval were obtained from the Human Ethics Committees at Massey University and the University of Canterbury.

**Table 1:** Health Management Resources for Building Disaster Resiliency

**Construct**	**Domain or Category**	**Measure or Specific attribute**
**Health**-**Protective attitudes**^**a**^		
Health-related quality of life	Physical health status	SF12 (v1) [94]
	Mental health status	SF12 (v1) [94]
	Emotional well-being	SOC13 [95]
	Spiritual well-being	SS20 [96,97]
	Social well-being	FS [98]
Global quality of life	Global well-being	SWLS [99]
**Health**-**Protective behaviors**^**b**^		
Earthquake and tsunami evacuation preparedness	Talking about these events	With social network
		With neighborhood
	Seeking information	Risks and consequences
		How to prepare
		How to respond
		How to evacuate
	Making evacuation plans	Survival and escape
		Evacuation and dislocation
		Communication
	Testing evacuation plans	Evacuation route
		Assembly area
		Participated in drill
	Making disaster kits	Survival and escape
		Evacuation and dislocation
		Communication
		Kit accessible

*Abbreviations*: *FS* 6-item Friendship Scale, *SF*12 12-item Short Form Health Survey, *SOC*13 13-item Sense of Coherence scale, *SS*20 20-item Serenity Scale, *SWLS* 5-item Satisfaction With Life Scale.

^a^Defined as health-related quality of life and subjective well-being.

^b^Defined as disaster preparedness.

**Figure 1:**
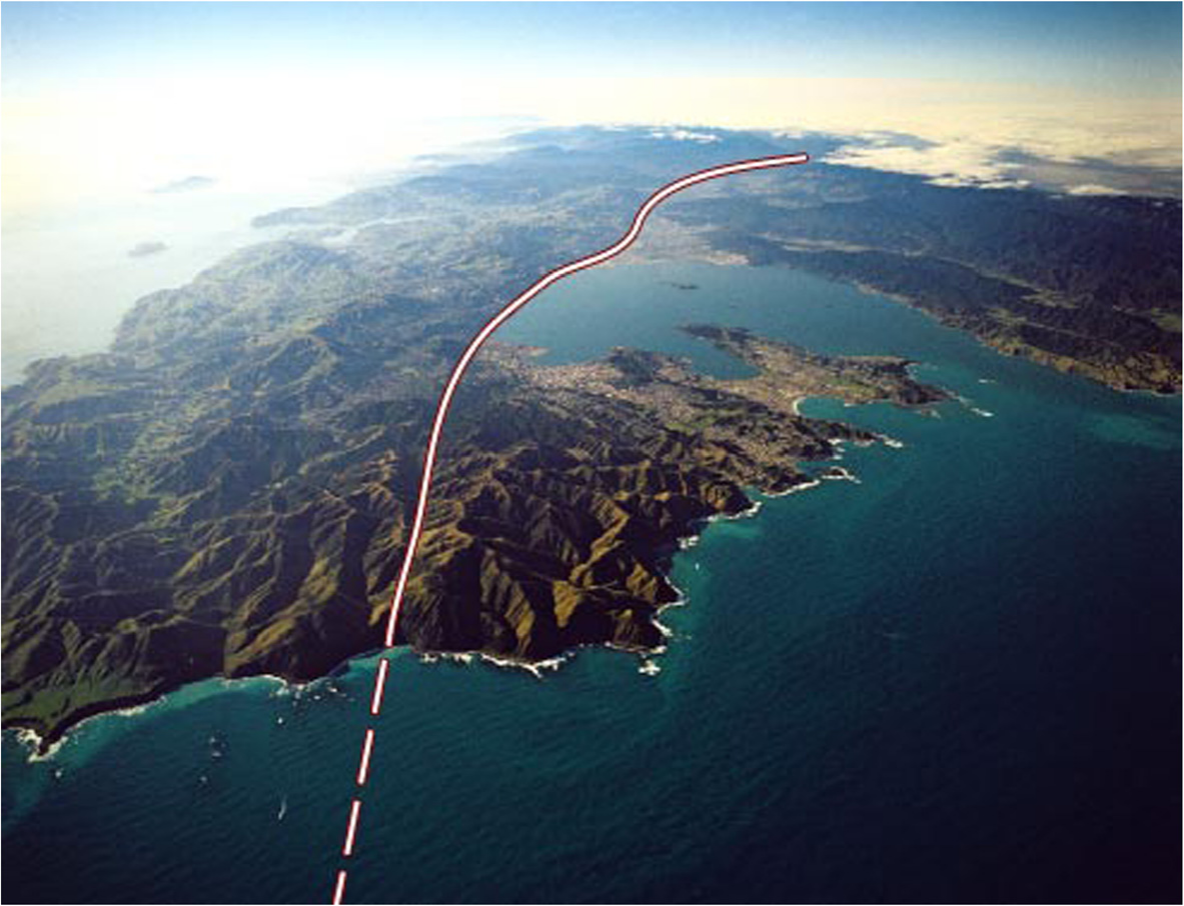
**Aerial views of the Wellington Region, Lower North Island, New Zealand.** The Wellington Fault is indicated by the dashed and solid line. The study area is to the east (right) of the Wellington Fault and consists of the elongate peninsula extending into Wellington’s Inner Harbour and out into Cook Strait, the isthmus connecting the peninsula to the lower North Island, and the ridgeline that adjoins the isthmus. (Original photographic image by Lloyd Homer, with Wellington Fault overlay provided courtesy of Jim Cousins, PhD, and image license granted by Margaret Low, GNS Science, Lower Hutt, New Zealand. Used with permission).

Administrative economy dictated a multistage cluster sampling plan. An area-based sampling frame was selected within an isolated physiographic region of eastern Wellington (Figure 1). This area, located east of the Wellington Fault, has a broad range of natural hazards (e.g., earthquake, tsunami, seiche, liquefaction, landslide, and wildfire). The potential for disruption of public utilities and loss of road access is high after a Wellington Fault earthquake [24-28], and the need for spontaneous or mandatory mass evacuations and domestic displacement from earthquake and tsunami [28,107-109] is conceivably greater here than elsewhere in Wellington.

A sample of 6 suburbs from the area-based frame was selected as the accessible population. Collectively, they encompassed various geographic, geologic, and socioeconomic conditions. A household was the unit of observation. The sample frame was all households within the boundaries of the 2006 Statistics New Zealand census maps for the 6 suburbs. The sample list was developed by using a systematic sampling interval plan of recording addresses for every other household on every street within the defined boundaries. Variations in accessibility (e.g., because of topography, building security, vacant or business properties) resulted in a final sampling interval of every second to third household and an accessible population of 2,451 residents. A probability sample was obtained for unit analysis of data at the level of 1 adult individual per household. Survey packets were posted in early November 2008 and the survey field period closed at the end of March 2009.

On-line sample size calculators [110] were used to determine the minimum recommended sample size for statistical similarity in population characteristics with a ±5% margin of error at a 95% confidence level. These calculations showed a need for 384 survey respondents; assuming a 30% response rate, this would require posting surveys to 1,277 households. We increased the total sample to 2,451 households to ensure sufficient data.

Five validated psychometric scales were selected to represent HrQoL domains, consistent with the WHO definition of health and QoL [76,77]. Physical health status and mental health status were measured using the Short Form Health Survey, 12-Item, version 1 [94]. Emotional well-being was measured using the 13-item Sense of Coherence scale [95,111]. Spiritual well-being was measured using the 20-item Serenity Scale [96,97]. Social well-being was measured using the 6-item Friendship Scale [98]. Global well-being was measured to address the WHO-QoL component of life satisfaction [76], using the 5-item Satisfaction with Life Scale [99]. We also asked participants to rate the perceived value of their health-management resources during a disaster evacuation on a 5-point Likert scale in each of the 5 HrQoL domains. To understand access to health care providers when self-management capacity is exceeded, we asked if they saw a regular general practitioner on at least an annual basis.

Many activities can promote disaster preparedness and organically translate into effective readiness. We identified 16 preparedness actions for earthquake and tsunami evacuation and grouped these items into 5 progressive levels of readiness (Table 1). People rated their level of engagement for each activity on a 5-point Stage of Change scale [112,113]. They were also asked to similarly rate their engagement in any other activity (unspecified) to increase their preparedness for any disaster type (presented as “other, please specify”).

People were further invited to describe (using unrestricted text) the 3 items they considered most essential for their personal evacuation kit.

Respondents were asked about their perceptions of disaster risk (type of threat; frequency of thought; imminence of threat) in rank order response format. People rated (on 5-point Likert scales) the perceived likelihood of various direct effects (property, health, and safety) and indirect effects (day-to-day life) of an earthquake or tsunami and their level of concern for required evacuation. Perceived preparedness to evacuate the home was measured with a single rating question item. To enrich our understanding of prior exposure to disaster, we sought anecdotal information (using open-ended questions) about the type of disaster experienced, personal impact, and the type of coping resources that were most helpful.

After obtaining these descriptive data, we asked 3 questions:

1. Were scores on HrQoL and life satisfaction associated with scores on evacuation kit activity?

2. Were scores on QoL and evacuation kit activity significantly different when stratified by age and gender?

3. How well did scores on HrQoL and life satisfaction explain scores on evacuation kit activity?

We evaluated relationships between scores on HrQoL and life satisfaction and scores on evacuation kit activity by using correlation coefficients. We tested for significant differences in scores on QoL and evacuation kit activity due to the effects of age and gender by using the *t* test, analysis of variance (ANOVA), or *χ*^2^ test, as appropriate. We used regression to determine how well HrQoL and life satisfaction scores explained scores on evacuation kit activity. For any proportion reported for the total sample, the results were accurate to within 3.8 percentage points with 95% confidence. For any mean reported for the total sample, results were accurate to within 3.8% of the standard deviation. For correlation coefficients calculated between 2 variables on the total sample, we had 80% power to detect a true correlation coefficient of 0.11 or greater. This was a small effect size [114], indicating that all but the smallest of correlation coefficients would be detected. Comparing 2 subgroups, equally split among 700 observations, provided a 2-sample *t* test with 80% power to detect a difference in mean scores of 21% times the SD (a small effect size [114]). It also provided an equality of proportions test with 80% power for a Fisher exact test to detect a difference of 11% between the proportions in each sample. The α level for all statistical tests was set at .05. No adjustment for multiple comparisons was made, given that this was an observational study and did not involve comparisons of control and intervention arms.

Qualitative string data were subject to content analysis, and responses were aggregated into summary categories and counts. They were subsequently tabulated and ranked from highest to lowest for all items selected by 10 or more respondents.

## Results

We received 695 survey responses (response rate, 28%). Age was normally distributed, with those aged 45 to 64 years making up the largest proportion (39%). The majority of respondents (62%) were women. The dominant ethnicity was New Zealand European (79%). A majority (77%) owned their home or were buying to live in it, had resided for more than 5 years in their suburb (63%) and in their current residence (54%), and lived in 1-family households (56%). A majority (75%) was employed at least part-time and had undergraduate (Level 7) degrees or higher (54%). A minority (34%) reported at least 1 dependent child.

Most respondents identified earthquakes and tsunamis as the hazards most capable of requiring evacuation in the Wellington Region, far exceeding perceived threats from any other threat. Most people contemplated these seismic events at least a few times a year (earthquake, 48%; tsunami, 58%). Two percent never thought about an earthquake occurring, and 21% never thought about a tsunami. Eighty-two percent recognized that an earthquake could trigger a disaster in Wellington within 10 to 100 years; 24% identified that an earthquake disaster could be within the next year (imminent). Thirty-two percent understood that a tsunami disaster could transpire within 10 to 100 years; 19% realized it could be imminent.

At least 1 in 3 people believed they were very likely to be adversely affected by an earthquake or tsunami in the Wellington Region; this covered a broad spectrum of direct effects (damage or loss of property, 48%; personal health and safety, 31%) and indirect effects (daily activities, 50%; mobility, 44%; level of support available from friends and family, 32%; income, 27%; evacuation requirements, 27%). Forty-five percent believed their property and daily life would be affected for more than 30 days. Twenty-one percent perceived that their health and safety would be affected for more than a month.

**Figure 2:**
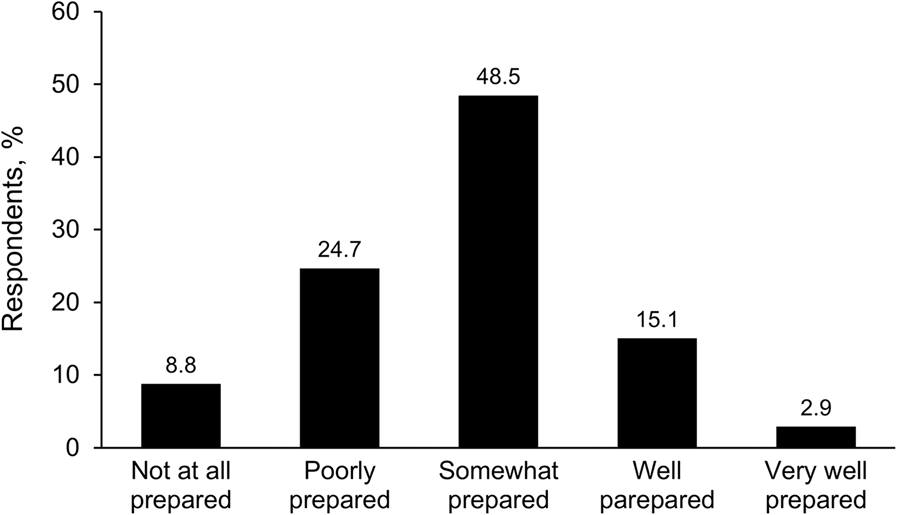
Perceived readiness for evacuation from home.

**Figure 3:**
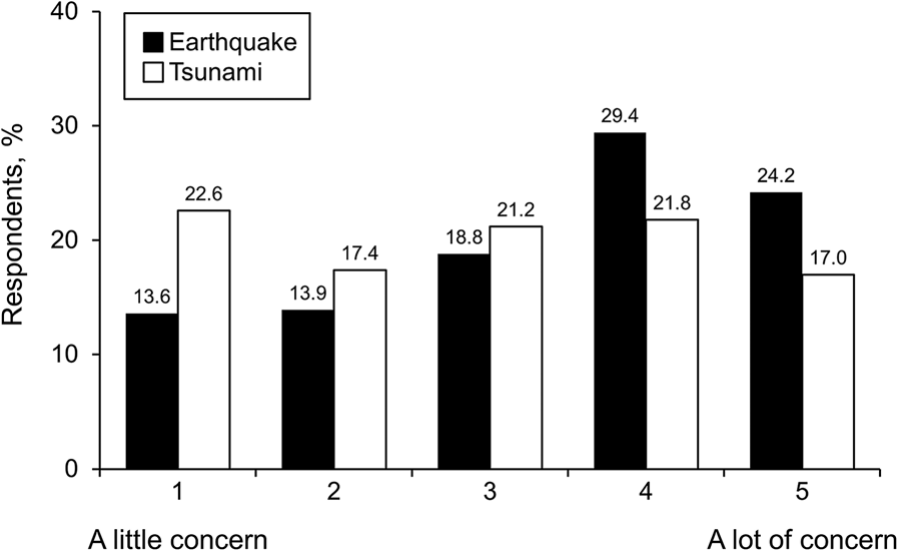
Level of concern for required evacuation.

**Figure 4:**
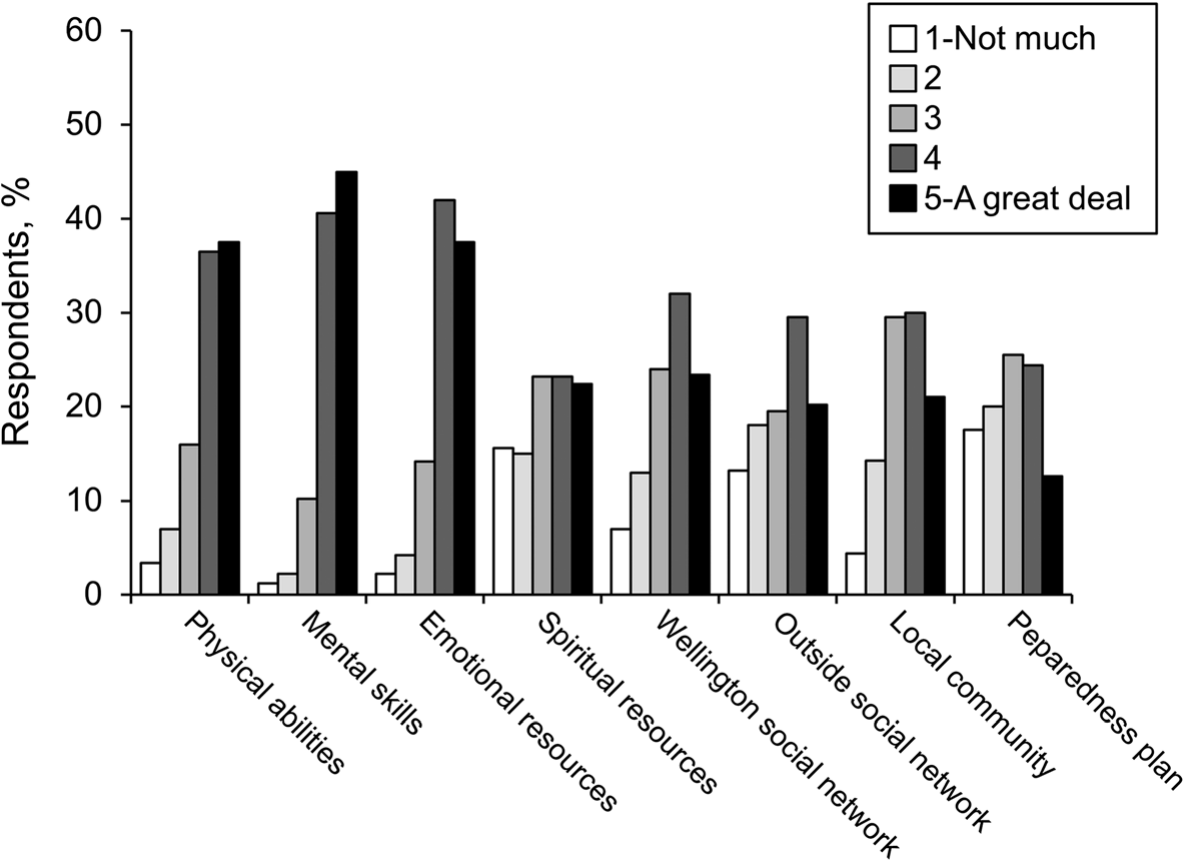
Perceived value of personal resources for coping with evacuation.

Eighteen percent perceived themselves as being well prepared for evacuation (Figure 2). Thirteen percent thought that their current preparedness plan would help a great deal. Fifty-six percent and 37% reported they had taken (unspecified) action for earthquake and tsunami preparedness, respectively. Anecdotally, the top 3 items reported as essential for a personal evacuation kit were food, clothing or outerwear, and medications (Table 2).Figure 3 shows the level of concern for evacuations; 24% had “a lot of concern” because of earthquake effects, and 17% shared a similar level of concern for tsunami evacuation. Eighty-eight percent of respondents had never evacuated for any reason. Seventy percent had no prior experience with earthquake or tsunami disaster, and 58% had no prior experience with any other disaster type.No personal health resource was perceived by a majority as helping a great deal with disaster evacuation (Figure 4). For those with prior disaster experience, however, social network support was anecdotally reported as the most helpful resource for coping with the disaster by a margin of 50% over mental and emotional support combined; only 1 person identified physical health as the factor that helped the most.

**Table 2:** Items Anecdotally Reported as Most Essential for Personal Evacuation Kit

**Item**	**n**
Food	118
Clothing, outerwear	95
Medications	88
Documents, identification, wallet	47
Medical supplies and devices	45
Water	27
Footwear	26
Cell phone	26
Family	25
Radio	16
Blankets, insulation, sleeping bag	11
Pets	10

Eighty-eight percent of respondents reported having at least annual visits with a general practitioner. Overall, we observed that the study population showed positive health and well-being (Table 3); compared with global population norms [115-120], people reported higher levels of well-being in every domain of HrQoL and global well-being. Only small to moderate differences in mean QoL scores were found among age groups, and small differences in mean QoL scores were found between genders.

**Table 3:** Quality of Life in the Study Population

**Quality**-**of**-**Life domain**	**Possible score**, **range**	**Sample score**	
		**Mean (SD)**	**Range**
Social well-being (FS)	0-24	20.4 (4.0)	3-24
Emotional well-being (SOC13)	13-91	68.8 (11.1)	31-91
Spiritual well-being (SS20)	20-100	68.7 (12.4)	34-100
Physical health status (SF12 PCS)	0-100	50.2 (9.4)	16-67
Mental health status (SF12 MCS)	0-100	51.3 (8.5)	16-67
Global well-being (SWLS)	5-35	25.1 (6.8)	5-35

*Abbreviations*: *FS* 6-item Friendship Scale, *SOC*13 13-item Sense of Coherence scale, *SS*20 20-item Serenity Scale, *SF*12 12-item Short Form Health Survey, *PCS* physical component summary, *MCS* mental component summary, *SWLS* 5-item Satisfaction With Life Scale.

We observed modest differences in evacuation kit activity within age groups, with those aged 18 to 24 years least likely to prepare and those 65 and older most likely to prepare. A *χ*^2^ test for independence confirmed that age and kit activity status were significantly associated, with a medium effect size, *χ*^2^ (3, n = 664) = 18.028; *P* ≤ .001; Cramer’s V = .165. The strength of this relationship was moderate. A *χ*^2^ test for independence confirmed that gender and kit activity status were not significantly associated, *χ*^2^ (1, n = 664) = .004; *P* = .95; φ = −.006.

**Figure 5:**
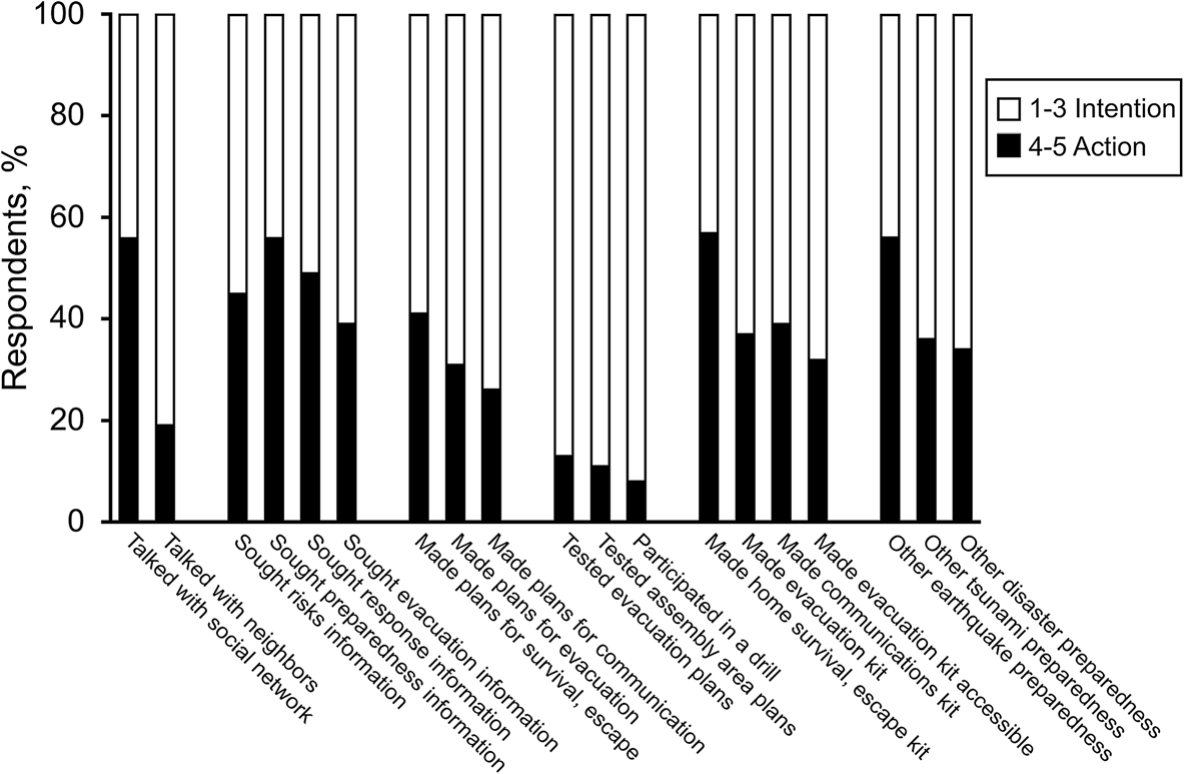
Level of engagement in disaster preparedness, stratified by intention vs action.

Examining the level of engagement in 16 preparedness activities (Table 1, health-protective behaviors), we observed slight differences between results observed at the Stages of Change level of measurement (Additional file 1: Table SA1) and under dichotomous conditions of intention vs action (Additional file 2: Table SA2). Figure 5 shows level of engagement in health-protective behaviors, stratified by intention vs action.

When examining relationships between mean QoL scores and evacuation kit activity, an independent samples *t* test showed differences in scores when comparing those who did and did not engage in preparedness, although differences here were modest. The magnitude of the observed effect (effect size) was small for emotional (.013) and spiritual (.011) well-being (Table 4). Correlation analyses showed weakly positive, significant associations (r = .10 to .29) for the domains of spiritual and emotional well-being (Table 5). Social and global well-being had slightly smaller effect sizes and weaker associations, and no significant effect was found for associations among physical and mental health status and evacuation kit activity (Tables 4, and 5). Similar correlations were found for other types of kit activity at the dichotomous level of measurement. “Made a communications kit” had weakly positive associations with emotional well-being (r = .14) and spiritual well-being (r = .13). Likewise, “Made a survival kit” was associated with social well-being (r = .13).

**Table 4:** Mean QoL Scores by Domain, Stratified by Evacuation Kit Activity Status

		**Intention**		**Action**			
**Quality**-**of**-**Life domain**	**Possible score, range**	**n**	**Score, mean (SD)**	**n**	**Score, mean (SD)**	***P *** **Value**^**a**^	**Effect Size**^**b**^
Social well-being (FS)	0-24	406	20.2 (4.3)	234	20.9 (3.6)	.047	.006
Emotional well-being (SOC13)	13-91	410	67.8 (11.3)	240	70.4 (10.2)	.004	.013
Spiritual well-being (SS20)	20-100	401	67.6 (12.8)	239	70.4 (11.4)	.006	.011
Physical health status (SF12 PCS)	0-100	403	50.6 (9.1)	239	49.7 (9.6)	.23	.000
Mental health status (SF12 MCS)	0-100	403	51.0 (8.6)	239	51.9 (8.1)	.19	.000
Global well-being (SWLS)	5-35	407	24.7 (6.8)	245	25.8 (6.7)	.03	.007

*Abbreviations*: *FS* 6-item Friendship Scale, *SOC*13 13-item Sense of Coherence scale, *SS*20 20-item Serenity Scale, *SF*12 12-item Short Form Health Survey, *PCS* physical component summary, *MCS* mental component summary, *SWLS* 5-item Satisfaction With Life Scale.

^a^Independent samples were compared by using a 2-tailed *t* test.

^b^Eta squared. Eta-squared is the proportion of total variation of the QOL domain attributable to the activity status. Guidelines for interpreting how much variance in kit activity can be accounted for by the scores from each QOL domain, and the importance of this effect size, are measured on a scale of 0 = no effect; .01 = small effect; .06 = moderate effect; .14 = large effect; 1 = perfect effect [114].

**Table 5:** Relationships Among QoL Domains and Evacuation Kit Activity Status

		**Stages of change**		**Dichotomized**	
**Quality-of-Life domain**	**n**	**Pearson *r***	***P *** **Value**^**a**^	**Pearson *r***	***P *** **Value**^**a**^
Social well-being (FS)	640	.07	.08	.08	.06
Emotional well-being (SOC13)	650	.12	.002	.11	.005
Spiritual well-being (SS20)	640	.15	<.001	.11	.006
Physical health (SF12 PCS)	642	−.08	.04	−.05	.23
Mental health (SF12 MCS)	642	.07	.08	.05	.19
Global well-being (SWLS)	652	.07	.08	.08	.03

*Abbreviations*: *FS* 6-item Friendship Scale, *SOC*13 13-item Sense of Coherence scale, *SS*20 20-item Serenity Scale, *SF*12 12-item Short Form Health Survey, *PCS* physical component summary, *MCS* mental component summary, *SWLS* 5-item Satisfaction With Life Scale.

^a^Two-tailed.

Within the 4 other categories of disaster preparedness (talking about earthquake or tsunami; seeking information about earthquakes or tsunamis; planning for disaster survival, evacuation, and communications; and testing evacuation plans), we observed weakly positive associations (r ≤ .18) with QoL, whether examined at the Stages of Change level (Additional file 3: Table SA3) or under dichotomized conditions of intention and action (Additional file 4: Table SA4). Again, the domains of well-being that consistently showed significant relationships with preparedness were spiritual, emotional, and global well-being.

Standard and hierarchical multiple regression analyses indicated that QoL variables, with and without inclusion of life satisfaction, explained 3.3% to 4.2% of variance (*P* < .01) in evacuation kit activity, as measured on the 5-level Stages of Change scale. Stepwise ordinary least squares regressions confirmed these results, with both models explaining 3.6% of variance in kit activity (*P* ≤ .001). When kit activity was dichotomized from 5 levels to 2 (intention vs action) and entered into a saturated logistic regression model, QoL variables explained from 4.6% to 6.8% of the variance (*P* ≤ .001). Stepwise logistic regressions provided results similar to the saturated logistic regression models (4.1%-5.8%; *P* ≤ .001), predicting that about 94% to 96% of the sample was in a state of intention rather than action, as explained solely by QoL variables.

When the effects of age and gender were entered into the regression models, we observed a significant but very small increase in how well the model predicted kit activity. The model as a whole explained 4.1% of variance (*P* ≤ .001). When life satisfaction was added, variance increased slightly to 4.2% (*P* = .001). Age and gender explained 1.9% of variance in kit activity (*P* = .002).

The strongest significant contribution to predicting kit activity was spiritual well-being (standardized regression coefficient β = .112; *P* = .01). The only other significant (but negative) factor was physical health status (β = −.085; *P* = .04). Results were consistent when life satisfaction was included. In the hierarchical analysis, the strongest significant factors (with and without life satisfaction) were spiritual well-being (β = .107; *P* = .01) and age (β = .097; *P* = .03). In logistic regression, the only significant factors were spiritual well-being (β = .015; *P* = .05) and age (β = 2.144; *P* = .006). When life satisfaction was included, only age was a significant contributor (β = 2.172; *P* = .005). In standard regression, spiritual well-being uniquely explained 1% of variance in kit activity. In hierarchical regression, spiritual well-being and age each uniquely explained less than 1% of the variance.

## Discussion

This large, population-based study yielded a data set with robust, reliable, representative, and generalizable results [88-92,121]. The sample size provided solid statistical power for interpreting the significance and relevance of the data. The response rate and cross-sectional nature of the study mandates that our findings be interpreted with care, and with appreciation of the epidemiologic and statistical axiom that “correlation does not imply causation”.

In summary, the data showed that Wellingtonians enjoy positive health and well-being, yet evacuation readiness was not prevalent. Associations between well-being and disaster preparedness were weak, but significant positive associations were observed for the domains of emotional and spiritual well-being and for life satisfaction. This fits within theoretical frameworks of QoL and salutogenesis that suggest health-protective attitudes and behaviors are positively related and that meaningfulness is a pivotal motivator [52-54,115,116].

Our finding that the majority of Wellington adults rated their overall QoL positively in each domain of well-being was an encouraging but perhaps unsurprising result. New Zealanders previously reported higher mean scores within physical and mental health domains compared with Australian and American populations [117-120], and the health status of Wellingtonians in our study was consistent with findings from recent national surveys [88-92]. Social well-being was further consistent with recent Australasian studies [118]. Levels of spiritual well-being were similar to means observed in other Wellington-based studies [96,97]. Emotional well-being was slightly greater than globally observed means [115,116]. Life satisfaction was as high or higher than means observed in developed countries [99,119,120]. The positive, health-oriented culture of New Zealand, with an emphasis on QoL, could have had a role in these results.

ANOVA indicated that the magnitude of the differences in mean QoL observed between age groups may not have any practical or theoretical significance. Differences (ie, the highest physical health and lowest mental health status in respondents 18–24 years old, and the converse for those aged 65 and older) are likely explained by the advantages of youth for positive physical health and the benefits of maturity toward all other domains of well-being. The slight degree to which gender was associated with higher mean QoL scores (higher mental health status in men, higher spiritual well-being in women) also may not have any significance. Within large sample sizes, some variance is natural, and even very small differences in groups can be statistically significant.

Notably, the data suggest that people generally were cognitively and affectively unprepared for a devastating earthquake and tsunami. This was a troubling discovery, further reinforced by our data indicating that half the survey respondents did not recognize the potential for being personally affected for greater than 30 days or even at all. The pervasiveness and persistence of these cognitive and affective gaps were underscored by data showing that only a small minority held considerable concern for evacuation due to earthquake (25%) or tsunami (20%) in this geographically isolated and exposed area. These outcomes were somewhat surprising, given that in the weeks preceding the field period for this study, a nationally broadcast television program (*Aftershock*, dramatizing an 8.2-magnitude earthquake and subsequent tsunami in Wellington [122]) received substantial media attention and public viewership. From a behavioral perspective, we emphasize that two-thirds had not made an evacuation plan, evacuation kit, or communications kit for earthquakes or tsunamis, and of those who had an evacuation plan, 90% had not tested the plan or shared it with someone outside the region.

Furthermore, apparent misunderstandings of how to prepare emerged. For example, food, clothing, and essential medicines emerged as the top 3 items (anecdotally reported) that were essential for evacuation kits, but identity documentation, medical prescriptions, and legal and financial papers were ranked about 50% less frequently, even though they are commonly identified as critical components of getaway kits in New Zealand [123-125]. Food and clothing tend to be easily replaceable in developed countries, whereas documentation and functional aids can be more crucial and more difficult and stressful to reclaim, reconstruct, or reacquire.

Importantly, overall risk perception and preparedness in New Zealand are now at the highest levels to date [126-129] because of the 2013–2014 seismicity in central New Zealand, the 2010–2013 Canterbury earthquakes, and the 2011 Tōhoku, Japan, earthquake and tsunami. However, these data and our results both show that the predominant focus has been on home survival (sheltering in place) for earthquake, rather than evacuation readiness or displacement. This orientation could be due to a tacit assumption of what event a person is anticipating: to survive an initial geophysical shock followed by a limited number of days in isolation or short-term displacement, rather than prolonged recovery or permanent relocation. Indeed, it can be difficult to anticipate and act on the unthinkable realities and consequences of seismic disasters.

Resistance to preparing for evacuation, changing behavior, and challenges in evacuation decision-making have long been noted [49,130,131]. Preparedness gaps observed here may further reflect contradictions in perceived vs actual preparedness, how “preparedness” and “readiness” are individually defined, or overconfidence regarding anticipated responses to new experiences. Considerable evidence suggests a lack of awareness, skepticism, denial, and false optimism about earthquake disaster in the Wellington Region [132-136]. Results from our quantitative, randomized sample (n = 695) also are broadly comparable with findings from a qualitative study (n = 400) compiled from interviews obtained through convenience sampling, on tsunami awareness and evacuation preparedness in the Wellington Region [137].

Fortunately, gaps in evacuation planning are receiving greater attention worldwide [138-141]. In New Zealand, the Crown Ministries and emergency management offices are increasingly integrating more tsunami risk assessment, evacuation planning, and preparedness messaging in disaster studies and plans [8,107-109,142-145]. This is essential for appropriate public responses when there is little or no time for warning or when environmental conditions necessitate spontaneous or forced displacement.

Our results also show that the nature and extent of the relationships between resilient well-being and disaster readiness are more complex and heterogeneous than previously acknowledged. Although no association was observed with gender, the regression models did detect small to moderate differences among age groups, with those aged 18 to 24 years least likely to prepare and those 65 and older most likely to prepare. These effects may reflect an acceptance of mortality and altruistic beneficence that increases with age; we observed that among respondents aged 65 years and older, many indicated motivation stemming from their health limitations or for the benefit of their loved ones, descendants, or posterity. The amount of heterogeneity among individuals, in terms of the well-being domains most strongly associated with preparedness, was striking. When examined on a domain-by-domain basis using validated and reliable scales, we showed significant, positive associations between engaging in evacuation preparedness and spiritual well-being (serenity), emotional well-being (strong sense of coherence), and global well-being (satisfaction with life).

Because this study is a novel application of QoL scales to a pre-event preparedness context, direct comparison with previous research is impossible. However, a telephone survey conducted in 14 American states from 2006–2010 (n = 104654), indicated that when comparing groups by the presence or absence of impaired physical and mental health and life satisfaction, those with impairments were more likely to have preparedness deficits [93]. Troubling gaps in evacuation readiness were also observed for those reporting physical and mental health impairments, and the authors highlighted a strong need for evacuation messaging [93].

Multiple studies of well-being and life satisfaction, conducted in the post-event context of various life adversities, strongly support our observations that different aspects of well-being are more salient and actionable at different ages and life circumstances and that all domains are important for overall positive health [80,146-151]. Further, perceived risk, HrQoL, and preparedness behavior often change organically after experiencing disaster, as do personal definitions of subjective well-being [152-155].

Prior disaster experience is likely to be among the strongest public motivators to prepare [81,82]. Post-disaster “windows of opportunity,” for survivors and those who experience disaster vicariously through events in the media, also motivate preparedness typically within a two-year period [82,156,157]. Consequently, for sustained readiness it is imperative to address both the pertinent social-cognitive constructs of preparedness messaging [48,81,82], and the decisional balance between intention and action that an individual encounters after receiving readiness cues [112,113,150].

The most effective interventions tend to target these distinct yet complementary influences [158]; the adoption and maintenance of health behaviors requires both motivation and volition [159]. For example, a decision to prepare might be confirmed by the collective behavior of a person’s social networks and community [48,82], but it also builds on the internal resolve a person has to create and act on intentions [80] – regardless of what other people are doing (or not doing).

Behavior is thus affected by and has an effect on multiple levels of influence [160,161]. Interventions that aim to affect the decisional balance to prepare – through personally-salient, intrinsically-motivating, and health-promoting beliefs and processes – are needed to better understand the strength of this personal component in preparedness. They may be important not only for building preparedness, but also for maintaining sustained readiness and resilience beyond “the teachable moment.”

Conducting an investigation is sometimes an intervention in itself, by raising self-awareness of what is personally important. In our own baseline data set, we observed disconnections between what people perceived as factors in their personal preparedness and what actually affected their preparedness actions. We note that the associations detected directly contrast with perceptions by the study population that physical and mental abilities are the most important QoL factors for disaster resilience and that spiritual resources are least helpful. Yet those respondents with personal disaster experience anecdotally reported that making meaning of the disaster and intentionally cultivating personal resilience helped them best cope. Although effect sizes are small, our quantitative data and analytic results were consistent with these anecdotal data.

Within the numerous explanatory models constructed to analyze our data set, overall QoL and well-being accounted for only 4.6% to 6.8% of the variability in evacuation preparedness, and spiritual well-being explained 2.2% of the variance. Although the latter is a small contribution, it was the strongest observed effect among all variables. Spiritual well-being may be an overlooked health-protective resource for building resiliency, effectively coping with adversity, and transforming the disaster experience into a meaningful or salutary life event.

There are many possible explanations for why the data showed these weak relationships. One is the presence of biases in risk perceptions [48-51,81,82,130-135]; the cognitive dissonance of logical inconsistencies abounds within our data set. These types of conundrums have been explored extensively by others to understand their etiology [50,162], develop better survey instruments [67,136], and improve risk communication strategies [163,164]. Secondly, as noted earlier, previous experience affects the frequency and structure of preparedness behavior [156,157]. Anecdotal reports by respondents indicated a significant number had survived a wide variety of prior disasters. Although many of these events cited dated to the mid- to late-20^th^ Century, the insights shared suggest these events have left a strong imprint on their risk perceptions.

A third alternative explanation is that a single, overall predictive model of disaster preparedness seems unlikely to exist; rather, a plethora of factors most likely explains preparedness as a whole, and the small percentage of the variance in preparedness activity from QoL and well-being reflects deeply personal approaches to wellbeing. The more the study variables were isolated in the analyses here, the less powerfully an effect was observed with preparedness. A result is that there is no evidence for making specific adaptations for preparedness education based on one particular domain of well-being at the expense of the other. Disasters are complex problems, with complex solutions, and disaster resilience is dynamic, personal, subjective, and sometimes situational.

Therefore, our data indicate a need for disaster preparedness programs that help people fully recognize their risk and build on their personal strengths and preferences (e.g., “*What makes me feel healthy*? *What does it take for me to be healthy*, *ready*, *and resilient*? *What will help me be resilient the most*? *What can I do to practice and become resilient in thought*, *belief*, *and action*? *What specific actions am I going to take to develop and follow my own personal resilience plan*, *in support of these outcomes*?”). By providing the opportunity for people to explore the multiple dimensions of quality of life and well-being, in the context of their own lives and health narratives, disaster researchers and practitioners can contribute to the development of truly “integrative disaster resilience.”

### Limitations

This study data may not be representative of all New Zealand, and results under current conditions may differ. Awareness and acceptance of Wellington’s disaster riskscape is higher now than when this survey was administered (2008–2009) because of more recent significant earthquakes in New Zealand (2010–2014) and the Samoa and Japan tsunamis (2009 and 2011, respectively).

We were unable to characterize nonrespondents to the survey, and they may be differently prepared than respondents. It also is impossible to predict whether a person will be at home when disaster strikes, if the evacuation kit will be accessible after damage to the home environment, or if a person’s current or future behavior will be consistent with indicated survey responses.

To our knowledge, this is the first application of the instruments used in this study in a pre-disaster setting, and thus we are unable to directly compare our results with other studies. The nature of the epidemiologic cross-sectional study design does not allow the interpretation of more than the strength and direction of relationships among variables and the contribution of an independent variable to explaining the variance in observed results.

## Conclusions

This study explored potential linkages and pathways for building resiliency in the general adult population of Wellington, New Zealand, by examining relationships between QoL and engagement in disaster preparedness. The data show that, despite above-average well-being and widespread hazard awareness, as a whole, people were not prepared for disaster and misperceived the nature of risk, readiness, and resilience. We found more similarities than differences in QoL, well-being, and preparedness, regardless of age and gender. Moreover, our data indicate that health-related QoL and global well-being were neither profound barriers to nor strong predictors of preparedness. The data, however, reveal a rarely considered dimension for building readiness: a sense of meaning was positively associated with preparedness and had the strongest positive effect among all the variables analyzed here. By nature of the study’s cross-sectional design, these observed results may vary over time and repeated sampling with higher response rates. Additional research could yield greater insight into the continuity or changes over time in these associations between well-being and preparedness.

All people need to prepare, and preparedness messages that address the layers of risk and resources for personal readiness and adaptation are vital. Preventive disaster research is valuable for finding effective pathways for building resilience. The evidence base contributed by this study indicates that interventions should generally focus on assisting individuals, rather than targeting specific groups; disaster consequences can easily overwhelm sociodemographic boundaries, and people have deeply personal expressions of resilient well-being. Certainly, tailored interventions will continue to be needed and important for those with heightened vulnerability to the compounding consequences of disaster. This research provides evidence, however, that everyone is vulnerable in Wellington, everyone needs greater engagement in evacuation planning and preparedness actions, and it is critical for everyone to think of being prepared, ready, and resilient as more than surviving.

Social and community groups and their sharing of preparedness information can facilitate program delivery and processing of these preparedness action messages [48,82]. Moreover, we further argue that delivering preparedness content with broad-brush strokes to the general population on how to be cognitively ready in thought (“*I have the fundamental awareness that the threat is present*, *and it can happen to me*”); affectively ready in belief (“*feelings are natural reactions to loss*, *and I have multidimensional strengths and resources to manage them*”); and behaviorally ready in action (“*I am building my resources and capacities now to respond and positively adapt for my own unique situation*) can additionally promote integrative resilience in all timeframes – prior to, during, and post-disaster.

Clearly, many continuums of comprehension, acceptance, and action overlap within a given community. All disasters are “local”; therefore, solutions must make fundamental sense, must reflect salient dimensions of physical, mental, emotional, social, spiritual, and global well-being for the residents, and must be relevant to their riskscapes.

Preventive health models [165-170] are natural vehicles for promoting readiness. They also complement emergency management policies and practices. Coupling these two well-established paradigms in New Zealand can create conditions that help people build personal resources for facing future adversity and engaging meaningfully in preparedness. Empowering individuals to transcend readiness barriers by engaging in health-promoting actions can inspire greater resilient well-being. From these personal successes will arise community partnership opportunities and the potential to amplify the emergence of disaster prevention and resiliency from local to global levels.

## Abbreviations

ANOVA: Analysis of variance; DRR: Disaster risk reduction; HrQoL: Health-related quality of life; QoL: Quality of life; WHO: World Health Organization.

## Competing interests

The authors declare that they have no competing interests.

## Authors’ contributions

MEG conceived of the study, selected the design, developed the survey questionnaire, collected the sample data, coordinated the study and survey administration, performed all data entry, data handling, and statistical analyses, and drafted the manuscript. RCK and JAS participated in the conception of the study, its design and coordination, development of the survey instrument and sampling and analyses plans, data analyses, and in the writing of the manuscript. All authors read and approved the final manuscript.

## Authors’ information

MEG has a PhD in Health Sciences, a Master of Science in Geology, and a Postgraduate Certificate in Public Health Preparedness, Response, and Recovery. She is a licensed professional geologist and Fellow of the Geological Society of America. She is currently a Postdoctoral Research Fellow at Mayo Clinic. RCK has a PhD in Psychology and is an Associate Professor in Health Sciences at the University of Canterbury. JAS has a PhD in Mathematical Statistics and is a Professor of Oncology and Biostatistics at Mayo Clinic.

## Supplementary Material

Additional file 1: Table SA1 Type of preparedness activity and level of engagement (%), at Stages of Change level of measurement. Click here for file

Additional file 2: Table SA2 Type of preparedness activity and level of engagement (%), at dichotomized level of measurement. Click here for file

Additional file 3: Table SA3 Associations among health-related quality of life, subjective well-being, and preparedness activity (Stages of Change). Click here for file

Additional file 4: Table SA4 Associations among health-related quality of life, subjective well-being, and preparedness activity (intention vs action). Click here for file
