# Supplementary material for: Building resiliency: a cross-sectional study examining relationships among health-related quality of life, well-being, and disaster preparedness
Source: Health Qual Life Outcomes. 2014 Jun 9;12:85. doi: 10.1186/1477-7525-12-85 (PMC4062284; doi:10.1186/1477-7525-12-85)
Supplement: Additional file 1: Table SA1 — Type of preparedness activity and level of engagement (%), at Stages of Change level of measurement. [file 1477-7525-12-85-S1.doc]

**Table A1.** Level of Engagement in Preparedness Activity, Stages of Change Model

|  |  | **Level of Engagement in Preparedness Activity** | | | | | | | | | | | | | |  |  |  | |  |  | |
| --- | --- | --- | --- | --- | --- | --- | --- | --- | --- | --- | --- | --- | --- | --- | --- | --- | --- | --- | --- | --- | --- | --- |
| **Preparedness** | **Specific Preparedness** | **1**  **Precontemplation** | |  | **2**  **Contemplation** | |  | **3**  **Intention** | |  | **4**  **Action** | |  | **5**  **Maintenance** | |  | **Summary Statistics** | | | | | |
| **Category** | **Activity** | **n** | **%** |  | **n** | **%** |  | **n** | **%** |  | **n** | **%** |  | **n** | **%** |  | **Mode** | | **Mean** | **SD** | | **Skewness** |
| Talking | Talked with social network (n=672) | 82 | 12.2 |  | 178 | 26.5 |  | 35 | 5.2 |  | 257 | 38.2 |  | 120 | 17.9 |  | 4 | | 3.2 | 1.3 | | −0.285 |
|  | Talked with others in neighborhood (n=657) | 259 | 39.4 |  | 217 | 33 |  | 54 | 8.2 |  | 95 | 14.5 |  | 32 | 4.9 |  | 1 | | 2.1 | 1.2 | | 0.899 |
| Information seeking | Sought information on risks and consequences (n=663) | 102 | 15.4 |  | 198 | 29.9 |  | 60 | 9 |  | 200 | 30.2 |  | 103 | 15.5 |  | 4 | | 3 | 1.4 | | −0.007 |
|  | Sought information on preparedness (n=667) | 65 | 9.7 |  | 160 | 24 |  | 68 | 10.2 |  | 253 | 37.9 |  | 121 | 18.1 |  | 4 | | 3.3 | 1.3 | | −0.348 |
|  | Sought information on response (n=665) | 75 | 11.3 |  | 183 | 27.5 |  | 82 | 12.3 |  | 215 | 32.3 |  | 110 | 16.5 |  | 4 | | 3.2 | 1.3 | | −0.142 |
|  | Sought information on evacuation (n=665) | 86 | 12.9 |  | 205 | 30.8 |  | 110 | 16.5 |  | 179 | 26.9 |  | 85 | 12.8 |  | 2 | | 3 | 1.2 | | 0.075 |

**Table A1** (continued)

|  |  | **Level of Engagement in Preparedness Activity** | | | | | | | | | | | | | |  |  |  | |  |  | |
| --- | --- | --- | --- | --- | --- | --- | --- | --- | --- | --- | --- | --- | --- | --- | --- | --- | --- | --- | --- | --- | --- | --- |
| **Preparedness** | **Specific Preparedness** | **1**  **Precontemplation** | |  | **2**  **Contemplation** | |  | **3**  **Intention** | |  | **4**  **Action** | |  | **5**  **Maintenance** | |  | **Summary Statistics** | | | | | |
| **Category** | **Activity** | **n** | **%** |  | **n** | **%** |  | **n** | **%** |  | **n** | **%** |  | **n** | **%** |  | **Mode** | | **Mean** | **SD** | | **Skewness** |
| Planning | Made survival or escape plans (n=666) | 107 | 16.1 |  | 199 | 29.9 |  | 88 | 13.2 |  | 167 | 25.1 |  | 105 | 15.8 |  | 2 | | 3 | 1.3 | | 0.091 |
|  | Made evacuation or dislocation plans (n=665) | 121 | 18.2 |  | 203 | 30.5 |  | 130 | 19.5 |  | 141 | 21.2 |  | 70 | 10.5 |  | 2 | | 2.8 | 1.2 | | 0.244 |
|  | Made communications plans (n=666) | 125 | 18.8 |  | 225 | 33.8 |  | 139 | 20.9 |  | 126 | 18.9 |  | 51 | 7.7 |  | 2 | | 2.6 | 1.2 | | 0.359 |
| Testing plans | Tested plans—followed an evacuation route (n=659) | 310 | 47 |  | 188 | 28.5 |  | 70 | 10.6 |  | 54 | 8.2 |  | 37 | 5.6 |  | 1 | | 2 | 1.2 | | 1.164 |
|  | Tested plans—gone to an assembly area (n=663) | 337 | 50.8 |  | 185 | 27.9 |  | 67 | 10.1 |  | 38 | 5.7 |  | 36 | 5.4 |  | 1 | | 1.9 | 1.1 | | 1.356 |

**Table A1** (continued)

|  |  | **Level of Engagement in Preparedness Activity** | | | | | | | | | | | | | |  |  |  | |  |  | |
| --- | --- | --- | --- | --- | --- | --- | --- | --- | --- | --- | --- | --- | --- | --- | --- | --- | --- | --- | --- | --- | --- | --- |
| **Preparedness** | **Specific Preparedness** | **1**  **Precontemplation** | |  | **2**  **Contemplation** | |  | **3**  **Intention** | |  | **4**  **Action** | |  | **5**  **Maintenance** | |  | **Summary Statistics** | | | | | |
| **Category** | **Activity** | **n** | **%** |  | **n** | **%** |  | **n** | **%** |  | **n** | **%** |  | **n** | **%** |  | **Mode** | | **Mean** | **SD** | | **Skewness** |
|  | Tested plans—participated in a drill (n=656) | 372 | 56.7 |  | 168 | 25.6 |  | 62 | 9.5 |  | 26 | 4 |  | 28 | 4.3 |  | 1 | | 1.7 | 1.3 | | 1.592 |
| Making kits | Made survival or escape kit (n=671) | 70 | 10.4 |  | 131 | 19.5 |  | 86 | 12.8 |  | 217 | 32.3 |  | 167 | 24.9 |  | 4 | | 3.4 | 1.3 | | −0.425 |
|  | Made evacuation kit (n=668) | 106 | 15.9 |  | 181 | 27.1 |  | 132 | 19.8 |  | 159 | 23.8 |  | 90 | 13.5 |  | 2 | | 2.9 | 1.3 | | 0.084 |
|  | Made communications kit (n=671) | 119 | 17.7 |  | 165 | 24.6 |  | 118 | 17.6 |  | 167 | 24.9 |  | 97 | 14.4 |  | 4 | | 2.9 | 1.3 | | 0.033 |
|  | Made kit accessible (n=668) | 139 | 20.8 |  | 117 | 17.5 |  | 133 | 19.9 |  | 110 | 16.5 |  | 105 | 15.7 |  | 2 | | 2.8 | 1.4 | | 0.25 |

**Table A1** (continued)

|  |  | **Level of Engagement in Preparedness Activity** | | | | | | | | | | | | | |  |  |  | |  |  | |
| --- | --- | --- | --- | --- | --- | --- | --- | --- | --- | --- | --- | --- | --- | --- | --- | --- | --- | --- | --- | --- | --- | --- |
| **Preparedness** | **Specific Preparedness** | **1**  **Precontemplation** | |  | **2**  **Contemplation** | |  | **3**  **Intention** | |  | **4**  **Action** | |  | **5**  **Maintenance** | |  | **Summary Statistics** | | | | | |
| **Category** | **Activity** | **n** | **%** |  | **n** | **%** |  | **n** | **%** |  | **n** | **%** |  | **n** | **%** |  | **Mode** | | **Mean** | **SD** | | **Skewness** |
| General preparedness (unspecified activity) | Taken steps for earthquake preparedness (n=670)  Taken steps for tsunami preparedness (n=650) | 55  127 | 8.2  19.5 |  | 144  191 | 21.5  29.4 |  | 93  94 | 13.9  14.5 |  | 258  160 | 38.5  24.6 |  | 120  78 | 17.9  12 |  | 4  2 | | 3.4  2.8 | 1.3  1.4 | | −0.406  0.174 |
|  | Taken steps for other disaster preparedness (n=133) | 28 | 21.1 |  | 41 | 30.8 |  | 19 | 14.3 |  | 19 | 14.3 |  | 26 | 19.5 |  | 2 | | 2.8 | 1.4 | | 0.319 |
