# Supplementary material for: Building resiliency: a cross-sectional study examining relationships among health-related quality of life, well-being, and disaster preparedness
Source: Health Qual Life Outcomes. 2014 Jun 9;12:85. doi: 10.1186/1477-7525-12-85 (PMC4062284; doi:10.1186/1477-7525-12-85)
Supplement: Additional file 2: Table SA2 — Type of preparedness activity and level of engagement (%), at dichotomized level of measurement. [file 1477-7525-12-85-S2.doc]

**Table A2.** Level of Engagement in Preparedness Activity, Stratified by Intention vs Action

|  |  | **Level of Engagement in Preparedness Activity** | | | | |
| --- | --- | --- | --- | --- | --- | --- |
| **Preparedness** | **Specific Preparedness** | **1-3 Intention** | |  | **4-5 Action** | |
| **Category** | **Activity** | **n** | **%** |  | **n** | **%** |
| Talking | Talked with social network (n=672) | 295 | 43.9 |  | 377 | 56.1 |
|  | Talked with others in neighborhood (n=657) | 530 | 80.7 |  | 127 | 19.3 |
| Information seeking | Sought information on risks and consequences (n=663) | 360 | 54.3 |  | 303 | 45.7 |
|  | Sought information on preparedness (n=667) | 293 | 43.9 |  | 374 | 56.1 |
|  | Sought information on response (n=665) | 340 | 51.1 |  | 325 | 48.9 |
|  | Sought information on evacuation (n=665) | 401 | 60.3 |  | 264 | 39.7 |
| Planning | Made survival or escape plans (n=666) | 394 | 59.2 |  | 272 | 40.8 |

**Table A2** (continued)

|  |  | **Level of Engagement in Preparedness Activity** | | | | |
| --- | --- | --- | --- | --- | --- | --- |
| **Preparedness** | **Specific Preparedness** | **1-3 Intention** | |  | **4-5 Action** | |
| **Category** | **Activity** | **n** | **%** |  | **n** | **%** |
|  | Made evacuation or dislocation plans (n=665) | 454 | 68.3 |  | 211 | 31.7 |
|  | Made communications plans (n=666) | 489 | 73.4 |  | 177 | 26.6 |
| Testing plans | Tested plans—followed an evacuation route (n=659) | 568 | 86.2 |  | 91 | 13.8 |
|  | Tested plans—gone to an assembly area (n=663) | 589 | 88.8 |  | 74 | 11.2 |
|  | Tested plans—participated in a drill (n=656) | 602 | 91.8 |  | 54 | 8.2 |
| Making kits | Made survival or escape kit (n=671) | 287 | 42.8 |  | 384 | 57.2 |
|  | Made evacuation kit (n=668) | 419 | 62.7 |  | 249 | 37.3 |

**Table A2** (continued)

|  |  | **Level of Engagement in Preparedness Activity** | | | | |
| --- | --- | --- | --- | --- | --- | --- |
| **Preparedness** | **Specific Preparedness** | **1-3 Intention** | |  | **4-5 Action** | |
| **Category** | **Activity** | **n** | **%** |  | **n** | **%** |
|  | Made communications kit (n=666) | 402 | 60.4 |  | 264 | 39.6 |
|  | Kit accessible (n=664) | 449 | 67.6 |  | 215 | 32.4 |
| General preparedness (unspecified activity) | Taken steps for earthquake preparedness (n=670)  Taken steps for tsunami preparedness (n=650) | 292  412 | 43.6  63.4 |  | 378  238 | 56.4  36.6 |
|  | Taken steps for other disaster preparedness (n=133) | 88 | 66.2 |  | 45 | 33.8 |
