# Supplementary material for: Building resiliency: a cross-sectional study examining relationships among health-related quality of life, well-being, and disaster preparedness
Source: Health Qual Life Outcomes. 2014 Jun 9;12:85. doi: 10.1186/1477-7525-12-85 (PMC4062284; doi:10.1186/1477-7525-12-85)
Supplement: Additional file 3: Table SA3 — Associations among health-related quality of life, subjective well-being, and preparedness activity (Stages of Change). [file 1477-7525-12-85-S3.doc]

**Table A3.** Associations Among Health-Related Quality of Life, Subjective Well-Being and Preparedness Activity (Stages of Change)

| **Type of Preparedness Activity** | | **Health-Related Quality of Life** | | | | |  |
| --- | --- | --- | --- | --- | --- | --- | --- |
|  |  | **Social Well-Being (FS)** | **Emotional Well-Being (SOC13)** | **Spiritual Well-Being (SS20)** | **Physical Health Status (SF12 PCS)** | **Mental Health Status (SF12 MCS)** | **Global Quality**  **of Life (SWLS)** |
| Talked with social network | Pearson’s *r*  *P* (2-tailed)  n | .11^a^  .007  642 | .09^b^  .019  654 | .06  .109  644 | .06  .162  647 | .06  .128  647 | .11^a^  .005  656 |
| Talked with others in neighborhood | Pearson’s *r*  *P* (2-tailed)  n | .09^b^  .026  630 | .11^b^  .005  652 | .16^c^  <.001  630 | −.08  .046  634 | .07  .085  634 | .10  .014  642 |
| Sought information on risks and consequences | Pearson’s *r*  *P* (2-tailed)  n | .10^b^  .014  634 | .13^a^  .002  645 | .14^c^  <.001  635 | .02  .564  638 | .10^a^  .008  638 | .14^c^  <.001  648 |
| Sought information on preparedness | Pearson’s *r*  *P* (2-tailed)  n | .11^b^  .011  640 | .14^c^  <.001  650 | .11^a^  .007  640 | .04  .304  643 | .14^c^  <.001  643 | .11^a^  .006  652 |

**Table A3** (continued)

| **Type of Preparedness Activity** | | **Health-Related Quality of Life** | | | | |  |
| --- | --- | --- | --- | --- | --- | --- | --- |
|  |  | **Social Well-Being (FS)** | **Emotional Well-Being (SOC13)** | **Spiritual Well-Being (SS20)** | **Physical Health Status (SF12 PCS)** | **Mental Health Status (SF12 MCS)** | **Global Quality**  **of Life (SWLS)** |
| Sought information on response | Pearson’s *r*  *P* (2-tailed)  n | .13^a^  .002  640 | .14^c^  <.001  648 | .13^a^  .001  640 | .04  .289  642 | .15^c^  <.001  642 | .11^a^  .005  650 |
| Sought information on evacuation | Pearson’s *r*  *P* (2-tailed)  n | .09^b^  .021  637 | .14^c^  <.001  648 | .18^c^  <.001  639 | 0  .981  642 | .12^a^  .002  642 | .12^a^  .002  650 |
| Made survival, escape plans | Pearson’s *r*  *P* (2-tailed)  n | .06  .110  637 | .11^a^  .006  649 | .13^a^  .001  639 | .01  .726  641 | .12^a^  .002  641 | .15^a^  <.001  650 |
| Made evacuation, dislocation plans | Pearson’s *r*  *P* (2-tailed)  n | .11^a^  .007  637 | .15^c^  <.001  647 | .12^a^  .002  638 | .02  .605  641 | .13^a^  .001  641 | .11^a^  .005  650 |

**Table A3** (continued)

| **Type of Preparedness Activity** | | **Health-Related Quality of Life** | | | | |  |
| --- | --- | --- | --- | --- | --- | --- | --- |
|  |  | **Social Well-Being (FS)** | **Emotional Well-Being (SOC13)** | **Spiritual Well-Being (SS20)** | **Physical Health Status (SF12 PCS)** | **Mental Health Status (SF12 MCS)** | **Global Quality**  **of Life (SWLS)** |
| Made communications plans | Pearson’s *r*  *P* (2-tailed)  n | .13^a^  .001  640 | .15^c^  <.001  649 | .15^c^  <.001  639 | .01  .784  643 | .11^a^  .005  643 | .15^c^  <.001  651 |
| Tested plans*—*followed an evacuation route | Pearson’s *r*  *P* (2-tailed)  n | .02  .679  630 | .03  .450  641 | .18^c^  <.001  634 | −.05  .255  635 | .10^b^  .012  635 | .09^b^  .020  643 |
| Tested plans—gone to an assembly area | Pearson’s *r*  *P* (2-tailed)  n | −.01  .743  633 | .01  .732  646 | .14^a^  .001  638 | −.08  .052  638 | .034  .397  638 | .06  .128  647 |
| Tested plans—participated in a drill | Pearson’s *r*  *P* (2-tailed)  n | .04  .348  627 | .03  .426  640 | .13^a^  .001  631 | −.031  .436  632 | .01  .844  632 | .06  .128  640 |

**Table A3** (continued)

| **Type of Preparedness Activity** | | **Health-Related Quality of Life** | | | | |  | |
| --- | --- | --- | --- | --- | --- | --- | --- | --- |
|  |  | **Social Well-Being (FS)** | **Emotional Well-Being (SOC13)** | **Spiritual Well-Being (SS20)** | **Physical Health Status (SF12 PCS)** | **Mental Health Status (SF12 MCS)** | **Global Quality**  **of Life (SWLS)** | |
| Made a survival, escape kit | Pearson’s *r*  *P* (2-tailed)  n | .11^a^  .007  642 | .09^b^  .028  653 | .03  .455  642 | .03  .511  645 | .06  .148  645 | .04  .308  655 | |
| Made an evacuation, dislocation kit | Pearson’s *r*  *P* (2-tailed)  n | .07  .078  640 | .12^a^  .002  650 | .15^c^  <.001  640 | −.08^b^  .042  642 | .07  .077  642 | .07  .079  652 | |
| Made a communication kit | Pearson’s *r*  *P* (2-tailed)  n | .07  .077  638 | .14^c^  <.001  648 | .16^c^  <.001  638 | −.049  .212  640 | .07  .066  640 | .08^b^  .036  650 | |
| Made kit accessible | Pearson’s *r*  *P* (2-tailed)  n | .06  .109  635 | .08  .051  646 | .12^a^  .002  639 | −.01  .721  641 | .04  .318  641 | | .10^a^  .009  649 |

**Table A3** (continued)

| **Type of Preparedness Activity** | | **Health-Related Quality of Life** | | | | |  | |
| --- | --- | --- | --- | --- | --- | --- | --- | --- |
|  |  | **Social Well-Being (FS)** | **Emotional Well-Being (SOC13)** | **Spiritual Well-Being (SS20)** | **Physical Health Status (SF12 PCS)** | **Mental Health Status (SF12 MCS)** | **Global Quality**  **of Life (SWLS)** | |
| Taken steps—earthquake preparedness | Pearson’s *r*  *P* (2-tailed)  n | .09^b^  .020  640 | .09^b^  .021  652 | .04  .282  643 | .03  .408  646 | .10^b^  .009  646 | | .09^b^  .016  655 |
| Taken steps—tsunami preparedness | Pearson’s *r*  *P* (2-tailed)  n | .09^b^  .019  622 | .10^b^  .010  633 | .15^c^  <.001  625 | 0  .945  626 | .13^a^  .001  626 | | .10^a^  .009  635 |
| Taken steps—other preparedness | Pearson’s *r*  *P* (2-tailed)  n | .07  .438  129 | .20^b^  .025  130 | .15  .085  128 | −.10  .293  122 | .137  .131  122 | | .19^b^  .029  129 |

Abbreviations: FS, Friendship Scale; SF12 MCS; 12-item Short Form Health Survey mental component summary; SF12 PCS, 12-item Short Form Health Survey physical component summary; SOC13, Sense of Coherence scale; SS20, Serenity Scale; SWLS, Satisfaction with Life Scale.

**Table A3** (continued)

^a^ *P* <.01

^b^ *P* <.05

^c^ *P* <.001
