# Supplementary material for: Building resiliency: a cross-sectional study examining relationships among health-related quality of life, well-being, and disaster preparedness
Source: Health Qual Life Outcomes. 2014 Jun 9;12:85. doi: 10.1186/1477-7525-12-85 (PMC4062284; doi:10.1186/1477-7525-12-85)
Supplement: Additional file 4: Table SA4 — Associations among health-related quality of life, subjective well-being, and preparedness activity (intention vs action). [file 1477-7525-12-85-S4.doc]

**Table A4.** Associations Among Health-Related Quality of Life, Subjective Well-Being, and Preparedness Activity (Intention vs Action)

| **Type of Preparedness Activity** | | **Health-Related Quality of Life** | | | | |  |
| --- | --- | --- | --- | --- | --- | --- | --- |
|  |  | **Social Well-Being (FS)** | **Emotional Well-Being (SOC13)** | **Spiritual Well-Being (SS20)** | **Physical Health Status (SF12 PCS)** | **Mental Health Status**  **(SF12 MCS)** | **Global Quality**  **of Life (SWLS)** |
| Talked with social network | Pearson’s *r*  *P* (2-tailed)  n | .13^a^  .001  642 | .09^a^  .029  654 | .05  .183  644 | .08^a^  .037  647 | .05  .179  647 | .12  .002  656 |
| Talked with others in neighborhood | Pearson’s *r*  *P* (2-tailed)  n | .07  .106  630 | .08^a^  .041  642 | .08^a^  .037  630 | −.04  .382  634 | .05  .181  634 | .12^b^  .004  642 |
| Sought information on risks and consequences | Pearson’s *r*  *P* (2-tailed)  n | .09^a^  .022  634 | .12^b^  .002  645 | .11^b^  .004  635 | .06  .129  638 | .08  .053  638 | .14^b^  .001  648 |
| Sought information on preparedness | Pearson’s *r*  *P* (2-tailed)  n | .10^a^  .015  640 | .14^c^  <.001  650 | .11^b^  .007  640 | .07  .078  643 | .14^c^  <.001  643 | .11^a^  .004  652 |

**Table A4** (continued)

| **Type of Preparedness Activity** | | **Health-Related Quality of Life** | | | | |  |
| --- | --- | --- | --- | --- | --- | --- | --- |
|  |  | **Social Well-Being (FS)** | **Emotional Well-Being (SOC13)** | **Spiritual Well-Being (SS20)** | **Physical Health Status (SF12 PCS)** | **Mental Health Status  (SF12 MCS)** | **Global Quality**  **of Life (SWLS)** |
| Sought information on response | Pearson’s *r*  *P* (2-tailed)  n | .11^b^  .007  640 | .14^c^  <.001  648 | .11^b^  .004  640 | .08  .057  642 | .14^c^  <.001  642 | .10^a^  .015  650 |
| Sought information on evacuation | Pearson’s *r*  *P* (2-tailed)  n | .08  .055  637 | .12^b^  .003  648 | .14^c^  <.001  639 | 0  .979  642 | .11^b^  .008  642 | .08^a^  .044  650 |
| Made survival, escape plans | Pearson’s *r*  *P* (2-tailed)  n | .06  .151  637 | .08^a^  .036  649 | .08^a^  .042  639 | .03  .453  641 | .11^b^  .006  641 | .13^b^  .001  650 |
| Made evacuation, dislocation plans | Pearson’s *r*  *P* (2-tailed)  n | .09^a^  .017  637 | .12^b^  .003  647 | .07  .089  638 | .02  .663  641 | .14^b^  .001  641 | .06  .136  650 |

**Table A4** (continued)

| **Type of Preparedness Activity** | | **Health-Related Quality of Life** | | | | |  |
| --- | --- | --- | --- | --- | --- | --- | --- |
|  |  | **Social Well-Being (FS)** | **Emotional Well-Being (SOC13)** | **Spiritual Well-Being (SS20)** | **Physical Health Status (SF12 PCS)** | **Mental Health Status  (SF12 MCS)** | **Global Quality**  **of Life (SWLS)** |
| Made communications plans | Pearson’s *r*  *P* (2-tailed)  n | .11^b^  .006  640 | .12^b^  .002  649 | .11^b^  .005  639 | .04  .340  643 | .09  .017  643 | .11^b^  .005  651 |
| Tested plans*—*followed an evacuation route | Pearson’s *r*  *P* (2-tailed)  n | .018  .650  630 | .03  .481  641 | .10  .011  634 | −.04  .320  635 | .11^b^  .008  635 | .06  .127  643 |
| Tested plans—gone to an assembly area | Pearson’s *r*  *P* (2-tailed)  n | 0  .918  633 | .02  670  646 | .06  .145  638 | −.05  .214  638 | .02  .664  638 | .003  .942  647 |
| Tested plans—participated in a drill | Pearson’s *r*  *P* (2-tailed)  n | .05  .242  627 | .05  .210  640 | .05  .197  631 | −.032  .425  632 | .013  .742  632 | .04  .315  640 |

**Table A4** (continued)

| **Type of Preparedness Activity** | | **Health-Related Quality of Life** | | | | |  |
| --- | --- | --- | --- | --- | --- | --- | --- |
|  |  | **Social Well-Being (FS)** | **Emotional Well-Being (SOC13)** | **Spiritual Well-Being (SS20)** | **Physical Health Status (SF12 PCS)** | **Mental Health Status  (SF12 MCS)** | **Global Quality**  **of Life (SWLS)** |
| Made a survival kit | Pearson’s *r*  *P* (2-tailed)  n | .13^b^  .001  642 | .08^a^  .035  653 | −.01  .743  642 | .05  .195  645 | .06  .158  645 | .05  .186  655 |
| Made an evacuation kit | Pearson’s *r*  *P* (2-tailed)  n | .08  .057  640 | .11^b^  .005  650 | .11^b^  .006  640 | −.05  .233  642 | .05  .186  642 | .08^a^  .034  652 |
| Made a communication kit | Pearson’s *r*  *P* (2-tailed)  n | .09^a^  .020  638 | .14^c^  <.001  648 | .13^b^  .001  638 | 0  .957  640 | .06  .092  640 | .08^a^  .037  650 |
| Made a kit accessible | Pearson’s *r*  *P* (2-tailed)  n | .08  .058  635 | .09^a^  .026  646 | .07  .062  639 | −.03  .449  641 | .044  .262  641 | .08^a^  .036  649 |

**Table A4** (continued)

| **Type of Preparedness Activity** | | **Health-Related Quality of Life** | | | | |  |
| --- | --- | --- | --- | --- | --- | --- | --- |
|  |  | **Social Well-Being (FS)** | **Emotional Well-Being (SOC13)** | **Spiritual Well-Being (SS20)** | **Physical Health Status (SF12 PCS)** | **Mental Health Status  (SF12 MCS)** | **Global Quality**  **of Life (SWLS)** |
| Taken steps—earthquake preparedness | Pearson’s *r*  *P* (2-tailed)  n | .10^b^  .009  640 | .08^a^  .032  652 | .04  .262  643 | .036  .363  646 | .10^a^  .016  646 | .11^b^  .007  655 |
| Taken steps—tsunami preparedness | Pearson’s *r*  *P* (2-tailed)  n | .12^b^  .002  622 | .14^c^  0  633 | .13^b^  .001  625 | .01  .809  626 | .14^b^  .001  626 | .13^b^  .001  635 |
| Taken steps—other preparedness | Pearson’s *r*  *P* (2-tailed)  n | .11  .201  129 | .27^b^  .002  130 | .15  .095  128 | −.14  .126  122 | .17  .069  122 | .30^b^  .001  129 |

Abbreviations: FS, Friendship Scale; SF12 MCS; 12-item Short Form Health Survey mental component summary; SF12 PCS, 12-item Short Form Health Survey physical component summary; SOC13, Sense of Coherence scale; SS20, Serenity Scale; SWLS, Satisfaction with Life Scale.

**Table A4** (continued)

^a^ *P* <.05

^b^ *P* <.01

^c^ *P* <.001
